# Evaluation of an *HMGA2* variant contribution to height and basal insulin concentrations in ponies

**DOI:** 10.1111/jvim.16723

**Published:** 2023-05-06

**Authors:** Brianna L. Clark, Nicholas J. Bamford, Allison J. Stewart, Molly E. McCue, Aaron Rendahl, Simon R. Bailey, François‐René Bertin, Elaine M. Norton

**Affiliations:** ^1^ School of Veterinary Science The University of Queensland Gatton Queensland Australia; ^2^ Melbourne Veterinary School The University of Melbourne Parkville Victoria Australia; ^3^ College of Veterinary Medicine The University of Minnesota St Paul Minnesota USA; ^4^ College of Agricultural and Life Sciences The University of Arizona Tucson Arizona USA

**Keywords:** endocrinology, equine metabolic syndrome, genetics, hyperinsulinemia‐associated laminitis, insulin dysregulation, Shetland, Welsh pony

## Abstract

**Background:**

The *HMGA2*:c.83G>A variant was identified in Welsh ponies having pleiotropic effects on height and insulin concentration.

**Objective:**

Determine whether the *HMGA2*:c.83G>A variant is associated with decreased height and higher basal insulin concentrations across pony breeds.

**Animals:**

Two hundred thirty‐six ponies across 6 breeds.

**Methods:**

Cross‐sectional study. Ponies were genotyped for the *HMGA2*:c.83G>A variant and phenotyped for height and basal insulin concentrations. Stepwise regression was performed for model analysis using a linear regression model for height and mixed linear model for insulin with farm as a random effect. Coefficient of determination, pairwise comparison of the estimated marginal means and partial correlation coefficients (parcor) were calculated to assess the relationship between *HMGA2* genotype and height or insulin.

**Results:**

Breed and genotype accounted for 90.5% of the variation in height across breeds, and genotype explained 21% to 44% of the variation within breeds. Breed, genotype, cresty neck score, sex, age, and farm accounted for 45.5% of the variation in insulin, with genotype accounting for 7.1%. The *HMGA2* A allele frequency was 62% and correlated with both height (parcor = −0.39; *P* < .001) and insulin (parcor = 0.22; *P* = .02). Pairwise comparisons found A/A ponies were >10 cm shorter than other genotypes. Compared with G/G individuals, A/A and G/A individuals had 4.3 μIU/mL (95% confidence interval [CI]: 1.8‐10.5) and 2.7 μIU/mL (95% CI: 1.4‐5.3) higher basal insulin concentrations, respectively.

**Conclusions and Clinical Importance:**

These data demonstrate the pleiotropic effects of the *HMGA2*:c.83G>A variant and its role in identifying ponies at increased risk for insulin dysregulation.

**Abbreviations**
BCS: body condition score
CNS: cresty neck score
EMM: estimated marginal means
EMS: equine metabolic syndrome
HAL: hyperinsulinemia‐associated laminitis
HI: hyperinsulinemia
*HMGA2*
: high‐mobility group AT‐hook 2
ID: insulin dysregulation

## 1 INTRODUCTION

Equine metabolic syndrome (EMS) is a clustering of risk factors for hyperinsulinemia‐associated laminitis (the most common cause of laminitis^1^), a painful condition and the most frequently reported determinant for euthanasia among horse owners.^2^ The cause of EMS is multifactorial with a complex interplay of genetics and environment.^3^, ^4^ Insulin dysregulation (ID) and hyperinsulinemia‐associated laminitis are common in ponies. Therefore, validation of specific genetic risk factors is crucial for the identification of ponies at higher risk of ID before they develop clinical laminitis.^3^

A pony‐specific missense variant (c.83G>A) in the first exon of the *HMGA2* gene, originally identified as contributing to height in Shetland ponies,^5^ subsequently was identified as having pleiotropic effects on height and metabolic traits in a cohort of Welsh ponies.^6^ The A allele was found to be the major allele in this population with a frequency of 76% and was negatively correlated with height and positively correlated with basal insulin concentration, insulin concentration post‐oral sugar test, non‐esterified fatty acids concentration, and triglyceride concentration. The effect of the A allele on height was additive and its effects on the metabolic traits was recessive.

It is imperative that proposed genetic risk alleles are replicated and validated in independent populations. This is particularly important for complex genetic diseases where: (a) results could be influenced by unaccounted‐for risk factors, (b) differences in allele frequency or effect size can exist across populations, or (c) the variant effect is a result of the presence of unidentified modifying gene(s) by environmental interactions. It is also important to determine if the pleiotropic effect of *HMGA2*:c.83G>A is present across other pony breeds or unique to Welsh ponies.

Our objective was to evaluate the *HMGA2*:c.83G>A variant in a diverse population of ponies. We hypothesized that the *HMGA2* variant would have a similar allele frequency and be associated with both height and basal insulin concentration across multiple pony breeds.

## 2 MATERIALS AND METHODS

### 2.1 Animals

A total of 236 ponies (186 mares, 21 geldings and 29 stallions) with an average age of 15 years (range, 10‐35 years) were recruited from Victoria, Australia. Ponies were sampled across 33 farms and 6 breeds including Shetland (n = 120), Welsh (n = 66), Australian (n = 21), Australian Riding (n = 19), New Forest (n = 4) and Highland (n = 6) ponies. The Welsh breed is divided into 4 sections (A‐D), which are defined based on pedigree, conformation and height, creating distinct subpopulations that can lead to variations in allele frequencies among sections. Our cohort included 37 section A, 22 section B, 1 section C, 1 section D, and 5 Welsh‐cross ponies. For all ponies, phenotyping was performed in the spring. Signalment, laminitis status (current and historical), body condition score (BCS),^7^ cresty neck score (CNS),^8^ and height at the withers (cm) were recorded. To minimize confounders for hyperinsulinemia, ponies were excluded from enrollment if they had clinical signs of pituitary pars intermedia dysfunction (PPID) or spring plasma ACTH concentrations >40 pg/mL (Immulite 1000 Immunoassay System, Siemens Healthcare Pty Lt, Bayswater, Australia).^9^, ^10^, ^11^ The study was approved by the University of Melbourne Animal Ethics Committee, application 2015160.1.

### 2.2 Insulin

Ponies were allowed free choice access to pasture and hay, but high non‐structural feeds were withheld for at least 5 hours before basal blood samples as previously recommended.^3^ Pasture on all properties where ponies were kept was unimproved native pasture. Blood was collected from the jugular vein into silicone‐coated plastic serum collection tubes. Blood was allowed to clot at ambient temperature and then samples were placed on ice and centrifuged within 3 to 8 hours of sampling. The separated serum was stored at −80°C until processing. Insulin concentrations were measured using a chemiluminescent assay (Immulite 1000 Immunoassay System, Siemens Healthcare Pty Ltd, Bayswater, Australia) previously validated for use in horses.^12^, ^13^ The insulin assay had a lower and upper limit of detection of 2 and 300 μIU/mL, respectively.

### 2.3 Genotyping

The DNA was isolated from hair roots according to manufacturer instructions (Gentra Puregene Tissue Kit, Qiagen, Germantown, Maryland). Genotyping of *HMGA2*:c.83G>A was performed using a previously described TaqMan SNP genotyping assay (see Supplemental Methods for primers and PCR protocol in Appendix S1).^5^ Results from the genotyping assay were analyzed using BioRad's CFX Manager Software (version 3.1).

### 2.4 Statistical analysis

All statistical analyses were performed using the programming language R version 4.1.1.^14^ Height and insulin concentration were assessed for normality using quantile‐quantile plots. Height was normally distributed and insulin was log transformed. Model analysis was performed using a stepwise regression with height or insulin as the outcome variable and *HMGA2* genotype, age, sex, CNS, and BCS as predictor variables; a *P*‐value of <.25 was set for predictor variables to enter the model and <.05 to remain in the model. A standard linear regression was used for height whereas a mixed linear model was used for basal insulin concentrations to account for the random effect of farm.

The percentage of phenotypic variation (*R* ^2^) explained for height and insulin were calculated for each of the explanatory variables. For insulin concentrations, the marginal *R* ^2^ was calculated to estimate the variance explained by the fixed effects using the R software package partR2^15^ and the intra‐class coefficient was calculated to estimate the variance explained by the random effects using the R software package rptR.^16^ The conditional semi‐partial *R* ^2^ (i.e., the variance explained by the fixed and random effects where the variance explained by the random effect is variable and dependent on each fixed effect) is presented in Table S1. A total of 10 000 bootstraps were performed to obtain the 95% confidence interval (CI) for our estimates. To estimate the strength of the relationship between *HMGA2* genotypes and the phenotypic variables after adjusting for covariates, the partial correlation coefficient was calculated using Equation (1):

(1) $$\sqrt{\frac{1-\sigma_{\text{full}}^{2}}{\sigma_{\text{reduced}}^{2}}}$$

where $\sigma_{\text{full}}^{2}$ and $\sigma_{\text{reduced}}^{2}$ are the maximum likelihood estimates of the residual variances from the models with genotype and without genotype, respectively.

Estimated marginal means (EMM) and pairwise comparisons were performed using the R software package emmeans,^17^ with height or basal insulin concentration as the outcome variable, *HMGA2* genotypes as the predictor variable, and the corresponding fixed and random effects determined from model analysis. A Tukey corrected *P*‐value of < .05 was used for significance between pairwise comparisons.

Notably, our basal insulin concentration data were censored, with 45 horses at the lower limit (<2 μIU/mL) and 22 horses at the upper limit (>300 μIU/mL) of detection of the assay. To account for censored data, a Tobit regression was performed, and the results were nearly identical to the mixed linear model. Our rationale for presenting the mixed linear model is that the Tobit regression requires calculation of a pseudo *R* ^2^ which is not equivalent to an adjusted *R* ^2^ which can alter statistical interpretation and does not allow for calculation of the contribution of each predictor. The results of the EMM from the Tobit regressions are presented in Figure S1. Furthermore, because of relatively small sample sizes, the Australian, Australian Riding, New Forest, and Highland ponies were included in the full cohort analysis but were excluded from the individual breed analyses. The results for the within breed analyses for the Australian and Australian Riding ponies are presented in Table S2.

## 3 RESULTS

### 3.1 *HMGA2* genotyping and allele frequency

The *HMGA2* genotyping assay results are presented in Table 1. The A allele, with an overall frequency of 62%, was the major allele across all pony breeds, but differences were found in the distribution of *HMGA2* genotypes among breeds. The Shetland ponies had the widest range in *HMGA2* genotypes, with an A allele frequency of 40%, and more individuals that were heterozygous or homozygous for the G allele. The majority of Welsh ponies, Australian ponies and Australian Riding ponies were homozygous for the A allele. Differences in *HMGA2* genotype distribution also were found among Welsh pony sections (Table 1). The Highland and New Forest ponies were fixed or nearly fixed for the G allele.

**TABLE 1:** Results from the *HMGA2*c.83G>A genotyping assay in a cohort of 236 ponies from 33 different farms.

Breeds	G/G	G/A	A/A
Welsh (n = 66)	1	5	60
Welsh A (n = 37)	0	0	37
Welsh B (n = 22)	0	2	20
Welsh C and D (n = 7)	1	3	3
Shetland pony (n = 120)	43	58	19
Australian pony (n = 21)	1	3	17
Australian riding pony (n = 19)	1	3	15
Highland pony (n = 6)	6	0	0
New Forest pony (n = 4)	2	2	0
Total	54	71	111

*Note*: G is the wild‐type allele and A is the variant allele.

### 3.2 Association between *HMGA2* genotypes and height

Stepwise regression indicated that *HMGA2* genotype and breed were significantly associated with height and they therefore were included in the final model. The final model accounted for 90.5% (*P* < .001) of the phenotypic variation in height, with breed and *HMGA2* genotypes explaining 81% and 9.5% of that variation, respectively. After removing breed variation, *HMGA2* genotypes accounted for 54.9% of the remaining phenotypic variation in height. Within breeds, genotype explained 21% of the phenotypic variation in height in the Welsh ponies (*P* < .001) and 44% (*P* < .001) in the Shetland ponies.

**FIGURE 1:**
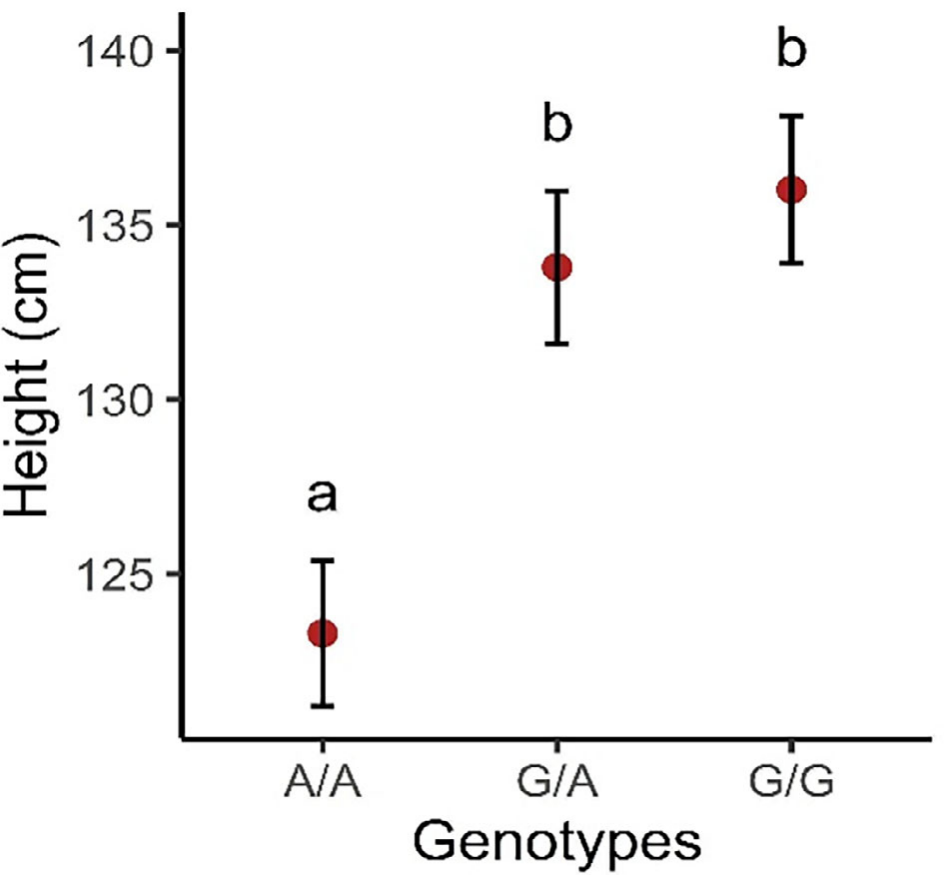
Estimated marginal means, 95% confidence intervals, and pairwise comparisons of the *HMGA2*:c.83G>A genotypes and height. Letters *a* and *b* indicate significant pairwise comparisons of the EMM with a Tukey corrected *P* < .05 between groups.

The partial correlation coefficient analysis identified a negative correlation (parcor = −0.39; *P* < .001) between genotype and height. Results for the EMM for genotype and height are presented in Figure 1. Pairwise comparisons of the EMM identified significant difference among genotypes, where A/A individuals were on average 10.4 cm shorter (95% CI: −12.4 to −8.32; *P* < .001) than G/A individuals and 12.6 cm shorter (95% CI: −14.9 to −10.2; *P* < .001) than G/G individuals. No significant difference was found between heterozygous and G/G individuals.

### 3.3 Association between *HMGA2* genotypes and basal insulin concentrations

Stepwise regression found that *HMGA2* genotype, age, sex, breed, and CNS were significantly associated with basal insulin concentrations and therefore were included as fixed effects in our model, with farm included as a random effect. Across all breeds, the model accounted for 45.5% of the phenotypic variation in insulin with 29.2%, 7.1%, 3.2%, 2.8%, 1.7%, and 1.5% of that variation being explained by farm, *HMGA2* genotype, sex, CNS, age, and breed, respectively (Table 2). Within the Welsh and Shetland ponies, *HMGA2* genotype accounted for 2.0% and 6.5% of the phenotypic variation in basal insulin concentrations, respectively (Table 2).

**TABLE 2:** Proportion of variance explained from *HMGA2* genotype, sex, cresty neck score (CNS), age, breed, and farm for basal insulin concentrations.

	All ponies	Welsh ponies	Shetland ponies
	(n = 236)	(n = 66)	(n = 120)
Genotype	0.071 (0.03‐0.18)	0.020 (0.01‐0.26)	0.065 (0.08‐0.19)
Sex	0.032 (0.01‐0.14)	0.012 (0.00‐0.26)	0.067 (0.08‐0.19)
CNS	0.028 (0.01‐0.14)	0.115 (0.08‐0.36)	0.011 (0.02‐0.14)
Age	0.017 (0.00‐0.13)	0.181 (0.15‐0.41)	0.00 (0.00‐0.13)
Breed	0.015 (0.00‐0.13)	—	—
Farm	0.292 (0.16‐0.49)	0.302 (0.08‐0.61)	0.320 (0.08‐0.55)

*Note*: Fixed effects were estimated using a marginal *R* ^2^ and farm was estimated using an intra‐class coefficient. A total of 10 000 bootstraps were performed to obtain the 95% confidence intervals.

**FIGURE 2:**
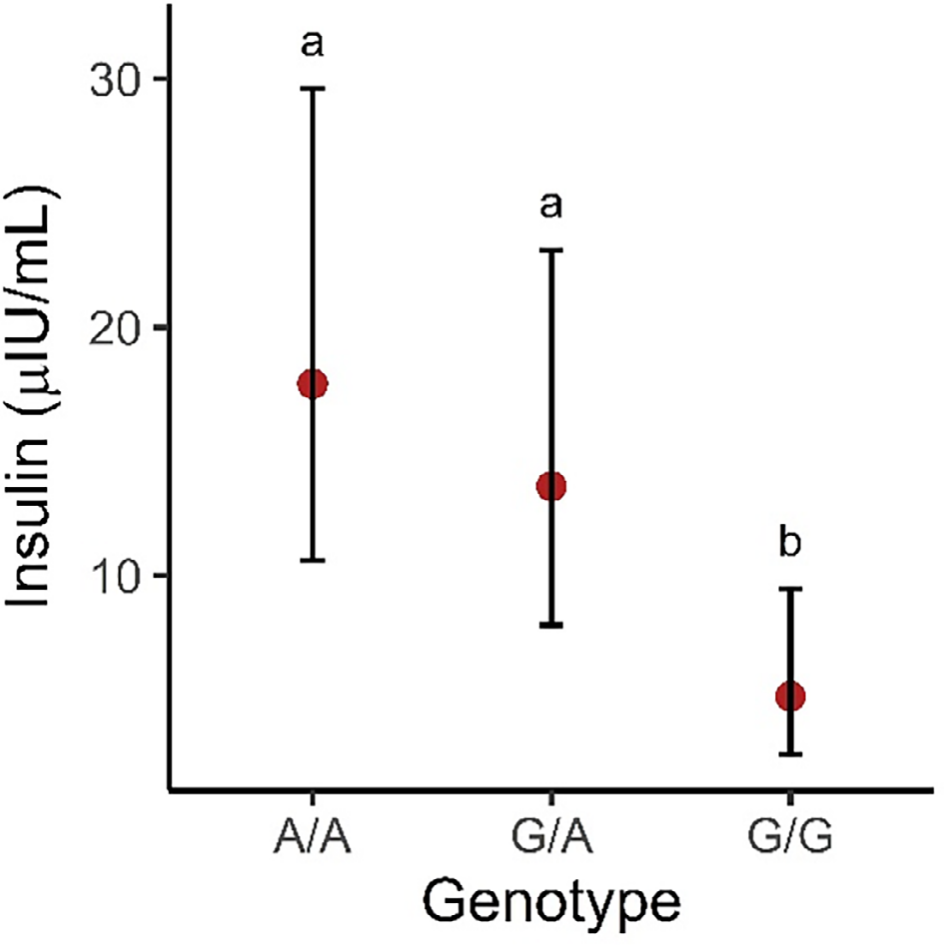
Estimated marginal means, 95% confidence intervals, and pairwise comparisons of the *HMGA2*:c83G>A genotypes and basal insulin concentration. Values have been back‐transformed to the original scale. Letters *a* and *b* indicate significant pairwise comparisons of the EMM with a Tukey corrected *P* < .05 between groups.

The partial correlation coefficient analysis identified a positive correlation (parcor = 0.22; *P* = .02) between *HMGA2* genotype and basal insulin concentration. The results of the EMM for *HMGA2* genotype and baseline insulin concentration are presented in Figure 2. Pairwise comparisons of the EMM identified significant differences among genotypes, where A/A and G/A individuals had higher basal insulin concentrations compared with G/G individuals by an average of 4.3 μIU/mL (95% CI: 1.8‐10.5; *P* < .001) and 2.7 μIU/mL (95% CI: 1.4‐5.3; *P* < .001), respectively. No significant difference was found between A/A individuals and heterozygotes (Figure 2).

## 4 DISCUSSION

We found that the A allele of the *HMGA2*:c.83G>A variant was present in 5 different pony breeds, was the major allele among the ponies sampled, and was correlated with height and basal insulin concentration. Individuals with at least 1 copy of the A allele had significantly higher insulin concentrations compared with G/G individuals, and A/A individuals were significantly shorter than both G/A and G/G individuals. After accounting for breed, the *HMGA2* variant contributed to over half of the remaining phenotypic variance in height and approximately 7% of the total variance in basal insulin concentration. These results support the *HMGA2* variant as having a pleiotropic effect on height and basal insulin concentrations in ponies.

Within our cohort, 47% of the population were homozygous for the A allele with an overall A allele frequency of 62%. The A allelic prevalence is comparable to other study cohorts of Shetland (approximately 75%) and Welsh (76%) ponies^5^, ^6^ and suggests that the A allele is the major allele across pony breeds. The *HMGA2*:c.83G>A variant mainly has been observed in ponies, with an A allele frequency of 0.5% reported in large breed horses.^5^ Here, we also identified notable differences in the distribution of *HMGA2* genotypes among breeds in our study. Although differences in allele frequency and variant effect size are expected across breeds, unequal sample sizes in our study also must be considered. In our population, most of the variation in *HMGA2* genotypes was driven by the Shetland ponies, which comprised approximately 50% of the population. A quarter of our population was Welsh ponies, with the majority being section A and B, which were nearly fixed for the A allele. Sections C and D, and Welsh‐cross ponies, which were previously found to have a higher prevalence of the G allele,^6^ were underrepresented in our cohort, which explains the decreased *HMGA2* genotype distribution observed in the Welsh breed overall. Both the Highland and New Forest ponies had the lowest prevalence of the A allele, with all Highland ponies homozygous for the G allele. Notably, these breeds tend to be taller compared with Shetland ponies or Welsh Section A and B ponies (Figure S2), which is consistent with a higher prevalence of the G allele, but only 4 to 6 ponies represented these breeds. Further investigation is required to confirm the population allele frequency for Highland, New Forest, Australian and Australian riding ponies.

We determined that the A allele was negatively correlated with height (parcor = −0.39), indicating that the more A alleles an individual had the shorter they tended to be. After accounting for breed, the *HMGA2* variant contributed to 54.9% of the remaining phenotypic variance in height. In accordance with earlier studies,^18^ this observation confirms that *HMGA2* is a major contributor to height in ponies. Notably, our EMM results suggested a recessive pattern of inheritance, whereas previous studies indicated an additive pattern (i.e., quantitative inheritance where each risk allele adds to the sum of the total effect).^5^, ^6^

The A allele was positively correlated (parcor = 0.22) with basal insulin concentration, indicating that the more A alleles an individual had, the higher the basal serum insulin concentration, which is similar to previous results.^6^ The *HMGA2* genotypes also accounted for 7.1% of the variation explained by the fixed effect (i.e., marginal variation) for insulin in our full cohort. Pairwise comparisons indicated a dominant effect of genotype on insulin concentration, where A/A and G/A individuals had higher basal insulin concentrations compared with G/G individuals. In contrast, a previous study identified a recessive pattern of inheritance between *HMGA2* and baseline insulin concentrations in Welsh ponies.^6^

Both height and basal insulin concentrations are complex genetic traits that have critical differences compared with simple (Mendelian) genetic traits. Complex genetic traits do not follow typical Mendelian inheritance patterns, often having an additive effect across multiple risk alleles, and the effect size of a single allele can be affected by other modifying alleles, which may be breed specific. The differences in mode of inheritance between *HMGA2* and height and insulin in our study compared with previous studies likely are explained by our heterogeneous cohort representing 6 different breeds with inherent breed variation and differences in allelic effect size. Importantly, to further understand the mode of inheritance, future studies should include larger sample sizes of each individual breed.

In complex genetic traits, >1 gene contributes to the phenotypic variation and genetic loci can have variable penetrance, expression and interaction with each other.^4^, ^18^, ^19^ Our results indicate that *HMGA2* has substantial impact on both height and insulin. However, the estimated allelic effect size of *HMGA2* on height was larger than that of insulin, supporting a previous study where HMGA2 explained 40% of the heritability of height and 20% of the heritability of basal insulin concentration.^19^ This conclusion is further supported by the literature where *HMGA2* is 1 of 4 genetic loci explaining 83% of the phenotypic variation in height in horses and ponies,^5^, ^6^, ^18^ indicating that height in horses is the result of a relatively small number of alleles with large effect sizes. Conversely, hyperinsulinemia has been shown to be highly heritable with hundreds of regions of the genome contributing to the phenotype.^20^ Thus, although as a single variant genetic test *HMGA2* may be useful for breeding decisions regarding height, it would not provide enough information to quantify a patient's total genetic risk for basal hyperinsulinemia. Hence development of a genetic test to predict EMS in at‐risk ponies would require a panel with moderate to high risk alleles identified across several breeds.

As complex genetic traits, both height and hyperinsulinemia manifest as a result of complex interactions between individual and environmental risk factors. Therefore, to ensure we accurately estimated the allelic effect size for *HMGA2*, we performed model analysis to identify potential covariates. For height, both breed and *HMGA2* remained in the model. Unsurprisingly, breed accounted for the majority of the phenotypic variation in height as the average height differs across pony breeds and was apparent in our cohort (Figure S2). When parsing the data among breeds, the *HMGA2* variant accounted for 21% of the variation in height in the Welsh ponies and 44% of the height variation in the Shetland ponies.

For basal insulin concentrations, previous studies have identified season,^21^, ^22^, ^23^ generalized obesity,^24^, ^25^, ^26^, ^27^, ^28^ regional adiposity,^24^, ^25^ diet,^28^, ^29^, ^30^, ^31^ exercise,^30^, ^31^, ^32^ age,^22^, ^24^ and genetics^19^, ^20^, ^33^ as risk factors for hyperinsulinemia. In our study, sampling was performed in the spring to control for season. The effect of sex on basal insulin concentrations is controversial but was considered in our model analysis by inclusion of mares, geldings, and stallions in our cohort.^24^, ^26^ Farm was included as a random effect to account for unknown environmental risk factors and non‐random sampling (farms were recruited in which both normal and hyperinsulinemic ponies could be sampled in the same geographic location with similar management strategies). Not surprisingly, farm was highly correlated with the fixed effects in our model (Table S1).

Stepwise regression indicated that farm, *HMGA2* genotype, CNS, breed, age, and sex were significantly associated with higher basal insulin concentrations and accounted for 45.5% of the variation in insulin. Body condition score did not remain in the final model, which is consistent with findings identifying CNS as more predictive of ID than BCS.^27^ Of the fixed effects (CNS, age, sex, breed, and *HMGA2* genotype), *HMGA2* genotype accounted for the largest overall percentage of the variation in basal insulin concentration. Farm accounted for the largest percentage of total variation in insulin, which is expected given the known contribution of the environment (i.e., diet and exercise) to basal insulin concentrations and hyperinsulinemia.^34^ Most farms in our study had a homogenous breed population, and the percentage variation in basal insulin concentrations explained by breed was decreased from 5% to 1.5% when accounting for the effect of farm. Notably, breed still was found to be significantly contributing to the phenotypic variation, suggesting differences in baseline insulin concentration among pony breeds. This finding is novel, with breed variation in insulin having been reported in large breed horses, and between horses and ponies, but not within pony breeds.^24^, ^35^ In the Shetland ponies, our model accounted for 46.3% of the total variation in basal serum insulin concentration, with the largest percentage being explained by sex and *HMGA2* genotype. This finding is not surprising because this cohort had the most diversity in *HMGA2* genotypes and the largest representation of stallions, which are thought to be more insulin sensitive.^36^ In the Welsh ponies, the model accounted for 63% of the variation in insulin concentration with the majority of the marginal variation being explained by age and CNS, and 2.0% of the variation was explained by *HMGA2* genotype. However, when excluding the section A Welsh ponies (fixed for the A allele), *HMGA2* genotype explained 21.1% (95% CI: 1.0%‐38%) of the variation in insulin. This finding suggests that the low variation in *HMGA2* genotypes in the Welsh pony cohort is underestimating the effect of *HMGA2* genotype. Notably, unequal sampling and the large amount of variation in basal insulin concentration within breeds also could explain the lack of significant differences observed between the pairwise comparisons of the EMM for breed and insulin (Figure S3). Thus, these data support evidence for breed variation in insulin concentration across pony breeds, but additional studies are required to further explore these findings and determine biological relevance.

Although our model (including the *HMGA2* genotype) documented a significant contribution to variation in insulin, a large proportion of the variation in insulin still was unexplained. This finding was expected because we have yet to identify all of the individual, environmental, and genetic risk factors contributing to ID. The primary objective of our study was to evaluate the pleotropic effect between *HMGA2* genotype and basal insulin concentrations. Thus, we did not explore other biochemical measurements associated with EMS nor did we perform dynamic testing for ID. Measuring baseline insulin concentration as an assessment for ID has poor sensitivity, but excellent specificity, compared with performing dynamic assessment of hyperinsulinemia after oral carbohydrate challenge.^3^ Future studies should be performed to validate the effect of the *HMGA2* allele on other biochemical variables. In addition, we focused our evaluation on the *HMGA2* c.83G>A variant, but future studies could include exploration of additional height alleles for pleiotropic effects on ID, including novel variants in *HMGA2* that have been associated with height in Chinese Debau ponies.^37^

To conclude, we identified the *HMGA2*:c.83G>A variant in a heterogenous population of ponies, with breed variation in genotype frequency. We identified the allele contributing to basal insulin concentration with height in other pony breeds and we also strengthened the notion of the complex genetic and epidemiological nature of EMS in ponies. Additional genetic studies are necessary before the development of a genetic test for EMS risk can be considered.

## CONFLICT OF INTEREST DECLARATION

Authors declare no conflict of interest.

## OFF‐LABEL ANTIMICROBIAL DECLARATION

Authors declare no off‐label use of antimicrobials.

## INSTITUTIONAL ANIMAL CARE AND USE COMMITTEE (IACUC) OR OTHER APPROVAL DECLARATION

Approved by the University of Melbourne Animal Ethics Committee, application number 2015160.1 with signed owner consent obtained for all procedures.

## HUMAN ETHICS APPROVAL DECLARATION

Authors declare human ethics approval was not needed for this study.

## Supporting information


**Appendix S1.** Supporting Information.
