# Supplementary material for: Evaluation of an HMGA2 variant contribution to height and basal insulin concentrations in ponies
Source: J Vet Intern Med. 2023 May 6;37(3):1186–92. doi: 10.1111/jvim.16723 (PMC10229368; doi:10.1111/jvim.16723)

Supporting information

S1. TAQ Genotyping Assay

Forward (CTTCAGCCCAGGGACAAC) and reverse (AAGCAGCAGCAAGTCAGT) primers were used to amplify the targeted DNA segment, and fluorescent probes were added to the reaction to tag the wild-type G allele (5HEX/AG+A+A+GA+G+G+ACG/3IABkFQ) or variant A allele (FAM/AG+A+GA+G+A+A+CGC/3IABkFQ) for allelic discrimination. The PCR reaction mixture contained a final volume of 19.2 μL and included: 5 μL of DNA [5 ng/μL], 1 μL of forward primer, 1 μL of reverse primer, 0.1 μL of HEX probe, 0.1 μL of FAM probe, 5 μL of PrimeTime Gene Expression Master Mix, 4 μL betaine, and 3 μL molecular biology grade water. Cycling parameters were set at 40 repeat cycles with an annealing temperature of 95ºC.

Supplemental Table 1: The variance explained by both the fixed and random effect (conditional R^2) for basal insulin levels. The conditional R^2 estimates were calculated using the R software package partR2. These estimates indicate that the random and fixed effects were highly correlated. 1000 bootstraps were performed to obtain a 95% confidence interval

	**All Ponies (n=236)**	**Welsh Ponies (n=66)**	**Australian Riding Ponies (n=19)**	**Australian Ponies (n=21)**	**Shetland Ponies (n=120)**
Age	.31 (.18-.50)	.39 (.18-.65)	.21 (.00-.84)	.45 (.00-.90)	.27 (.08-.51)
Sex	.32 (.19-.51)	.22 (.00-.53)	.24 (.00-.85)	.42 (.00-.89)	.34 (.16-.57)
Geno	.34 (.22-.53)	.23 (.00-.53)	.20 (.00-.84)	.41 (.00-.88)	.34 (.15-.57)
CNS	.32 (.19-.51)	.33 (.11-.60)	.50 (.33-.95)	.48 (.06-.91)	.29 (.09-.52)
Breed	.29 (.16-.49)	-	-	-	-
Age+Sex	.33 (.21-.52)	.39 (.18-.65)	.24 (.00-.85)	.55 (.17-.93)	.35 (.16-.57)
Age+Geno	.36 (.23-.54)	.42 (.22-.67)	.22 (.00-.84)	.46 (.03-.90)	.34 (.16-.57)
Age+CNS	.33 (.20-.52)	.45 (.26-.67)	.53 (.35-.96)	.49 (.08-.91)	.29 (.09-.52)
Age+Breed	.31 (.17-.50)	-	-	-	-
Sex+Geno	.37 (.25-.55)	.24 (.00-.54)	.25 (.00-.85)	.43 (.00-.89)	.40 (.22-.61)
Sex+CNS	.34 (.21-.53)	.33 (.11-.60)	.51 (.33-.95)	.47 (.04-.90)	.35 (.16-.57)
Sex+Breed	.32 (.19-.51)	-	-	-	-
Geno+CNS	.37 (.25-.55)	.37 (.15-.63)	.51 (.33-.95)	.49 (.08-.91)	.35 (.16-.58)
Geno+Breed	.36 (,23-.54)	-	-	-	-
CNS+Breed	.32 (.19-.51)	-	-	-	-
Age+Sex+Geno	.37 (.25-.55)	.42 (.22-.67)	.25 (.00-.85)	.58 (.23-.94)	.41 (.22-.61)
Age+Sex+CNS	.35 (.22-.53)	.45 (.26-.69)	.54 (.36-.97)	.57 (.20-.93)	.35 (.16-.57)
Age+Sex+ Breed	.33 (.21-.52)	-	-	-	-
Age+Geno+ CNS	.38 (.26-.56)	.50 (.31-.72)	.54 (.36-.97)	.52 (.13-.92)	.36 (.17-.58)
Age+Geno+ Breed	.37 (.25-.55)	-	-	-	-
Age+CNS+ Breed	.33 (.20-.52)	-	-	-	-
Sex+Geno+ CNS	.39 (.27-.57)	.37 (.16-.63)	.51 (.34-.95)	.48 (.06-.91)	.40 (.22-.61)
Sex+Geno+ Breed	.38 (.26-.56)	-	-	-	-
Sex+CNS+ Breed	.34 (.22-.53)	-	-	-	-
Geno+CNS+ Breed	.39 .27-.56)	-	-	-	-
Age+Sex+ Geno+CNS	.39 (.27-.57)	.50 (.31-.72)	.56 (.38-.98)	.57 (.23-.94)	.41 (.23-.62)

Age+Sex+ Geno+Breed	.39 (.27-.56)	-	-	-	-
Age+Sex+ CNS+Breed	.35 (.22-.53)	-	-	-	-
Age+Geno+ CNS+Breed	.39 (.27-.57)	-	-	-	-
Sex+Geno+ CNS+Breed	.40 (.29-.56)	-	-	-	-
**Conditional R^2**	**.41 (.28-.58)**	**.50 (.31-.72)**	**.56 (.38-.98)**	**.57 (.23-.94)**	**.41 (.23-.62)**

Supplemental Table 2 : Proportion of variance explained from *HMGA2* genotype and Breed for height, and *HMGA2* genotype, sex, cresty neck score (CNS), age, breed and farm for basal insulin concentrations. For height, fixed effects were estimated using an R^2, and for basal insulin concentrations fixed effects were estimated using a marginal R^2 and farm was estimated using an intra-class coefficient. 10000 bootstraps were performed in order to obtain the 95% confidence intervals.

	**All Ponies (n = 236)**	**Welsh Ponies (n = 66)**	**Australian Riding Ponies (n = 19)**	**Australian Ponies (n = 21)**	**Shetland Ponies (n = 120)**
**Height**					
**Genotype**	0.095 (0.03-0.17)	0.21 (0.09-0.41)	0.32 (0.03-0.69)	0.36 (0.04-0.67)	0.44 (0.27-0.58)
**Breed**	0.81 (0.75-0.84)	-	-	-	-
**Basal Insulin Concentrations**					
**Genotype**	0.071 (0.03 - 0.18)	0.020 (0.01 - 0.26)	0.060 (0.00 - 0.44)	0.009 (0.00 - 0.47)	0.065 (0.08 - 0.19)
**Sex**	0.032 (0.01 - 0.14)	0.012 (0.00 - 0.26)	0.104 (0.00 - 0.46)	0.043 (0.00 - 0.51)	0.067 (0.08 - 0.19)
**CNS**	0.028 (0.01 - 0.14)	0.115 (0.08 - 0.36)	0.363 (0.10 - 0.57)	0.073 (0.00 - 0.54)	0.011 (0.02 - 0.14)
**Age**	0.017 (0.00 - 0.13)	0.181 (0.15 - 0.41)	0.066 (0.00 - .44)	0.043 (0.00 - 0.51)	0.00 (0.00 - 0.13)
**Breed**	0.015 (0.00 - 0.13)	-	-	-	-
**Farm**	0.292 (0.16 - 0.49)	0.302 (0.08 - 0.61)	0.299 (0.00 - 0.89)	0.494 (0.00 - 0.93)	0.320 (0.08 - 0.55)

Supplemental Figure 1: Estimated marginal means, 95% confidence intervals, and pairwise comparisons between basal insulin concentrations and breed using a Tobit regression. The model included the fixed effects of cresty neck score, age, sex, *HMGA2* genotype and the random effect of farm. 45 values were left censored (fell below the level of detection) and 22 values were right censored (fell above the upper limit of detection). Insulin values were back transformed from the log scale. Letters *a*, *and b* indicate significant pairwise comparisons of the EMM with a Tukey corrected $P < .05$ between groups

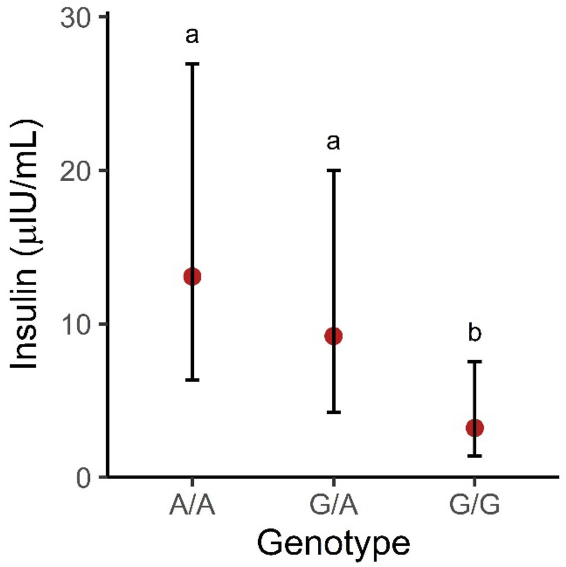

Pairwise Comparison	Ratio	Standard Error	P-Value
**A/A - G/A**	1.42	0.63	0.71
**A/A – G/G**	4.07	2.07	0.02
**G/A – G/G**	2.86	1.20	0.04

Supplemental Figure 2: Estimated marginal means (EMM), 95% confidence intervals, and pairwise comparisons between height and breed. Breeds included Australian ponies (Australian), Highland ponies, New Forest ponies, Australian Riding ponies (Riding), Shetland ponies, and Welsh ponies. There were no statistically significant differences identified between breeds. Letters *a*, *b, c and d* indicate significant pairwise comparisons of the EMM with a Tukey corrected $P < .05$ between groups. Notably, when separating out the section A Welsh ponies, they were statistically different than all other pony groups, and were on average 11-17cm shorter than the Australian, Highland, New Forest, and Riding ponies and ~27cm taller than the Shetland ponies (data not shown).

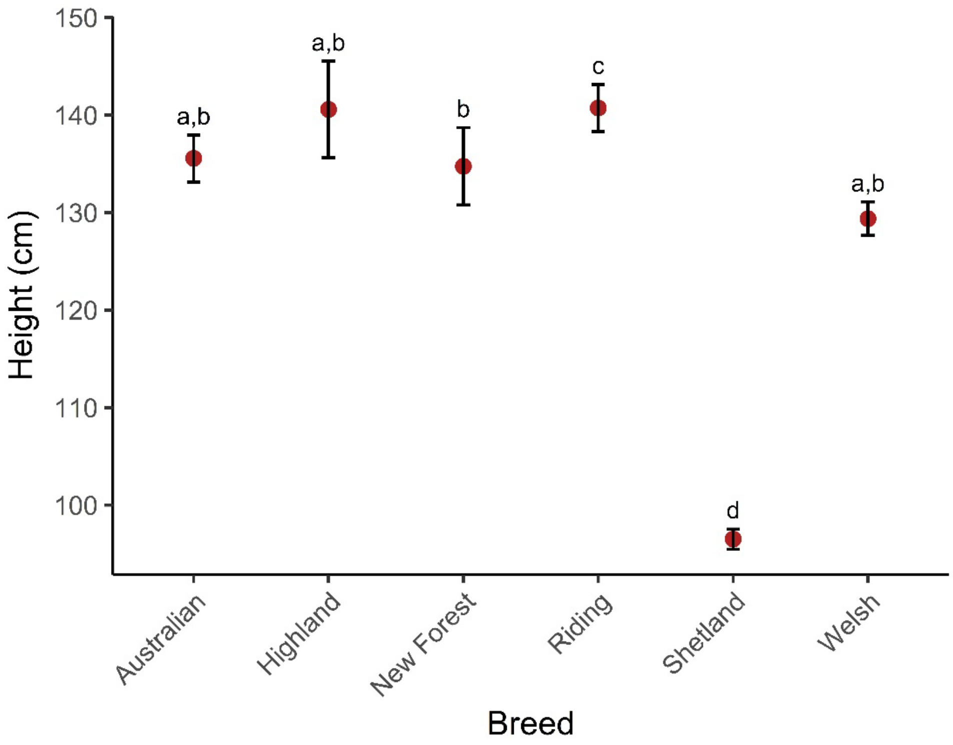

Supplemental Figure 3: Estimated marginal means, 95% confidence intervals, and pairwise comparisons between basal insulin concentrations and breed. The model included the fixed effects of cresty neck score, age, sex, *HMGA2* genotype and the random effect of farm. Breeds included Australian ponies (Australian), Highland ponies (Highland), Australian Riding ponies (Riding), Shetland ponies, and Welsh ponies. Insulin values were back transformed from the log scale. There were no statistically significant differences identified between breeds.

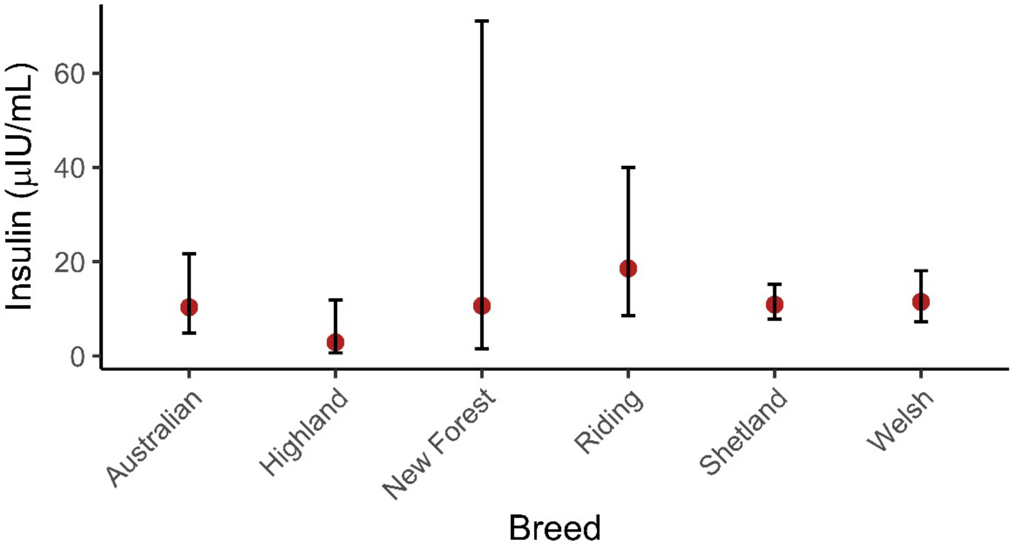

Supplement: Supplementary file 1 — Appendix S1. Supporting Information. [file JVIM-37-1186-s001.pdf]
